# The role of innate lymphoid cells and T helper cell activation in type 2 diabetic patients: a protocol for a systematic review and meta-analysis

**DOI:** 10.1186/s13643-019-1144-z

**Published:** 2019-09-03

**Authors:** Vuyolwethu Mxinwa, Tawanda M. Nyambuya, Phiwayinkosi V. Dludla, Bongani B. Nkambule

**Affiliations:** 1 School of Laboratory Medicine and Medical Sciences (SLMMS), College of Health Sciences, University of KwaZulu-Natal, Durban, South Africa; 2 Department of Health Sciences, Faculty of Health and Applied Sciences, Namibia University of Science and Technology, Windhoek, Namibia; 3 Department of Life and Environmental Science, Polytechnic University of Marche, Ancona, Italy; 4 Biomedical Research and Innovation Platform, South African Medical Research Council, Tygerberg, South Africa

**Keywords:** Hyperglycaemia, Glucose metabolism, Inflammation, Innate lymphoid cells, Natural killer cells, T helper cells, Anti-hyperglycemic

## Abstract

**Background:**

Persistent levels of low-grade inflammation and T lymphocyte activation are associated with insulin resistance and type 2 diabetes that eventually lead to the development of cardiovascular diseases. Interestingly, increasing studies report on an emerging role of innate lymphoid cells in the development of both type 2 diabetes and cardiovascular disease. This systematic review will provide a comprehensive synthesis of available studies reporting on the role of innate lymphoid cells and associated T helper cell function in type 2 diabetic patients. It will further provide insight into the association of innate lymphoid cell activation and cardiovascular risk in adults living with type 2 diabetes.

**Methods:**

This systematic review protocol has been prepared in accordance with Preferred Reporting Items for Systematic Review and Meta-Analysis Protocols 2015 guidelines. The protocol has been registered with PROSPERO (CRD42018106159). This systematic review and meta-analysis will include published randomised clinical trials, observational studies, and case-control studies. We will also include grey literature. A search strategy will be developed with the help of subject librarian using Medical Subject Heading (MeSH) words for MEDLINE. This will then be adapted for the Embase database. Two independent reviewers VM and BBN will screen all studies using prespecified inclusion and exclusion criteria. The Downs and Black checklist will be used to assess the quality of individual studies. Predefined relevant data items will be extracted using sheets, and all study tables will be created using Review Manager V.5.3. The Grading of Recommendations Assessment, Development and Evaluation approach will be used to assess the strength of evidence.

**Ethics and dissemination:**

The review will include publicly available data. The findings of this review will be disseminated through publications.

## Background

Bing overweight and obesity are existing global health problems, contributing significantly to the rapid rise in metabolic disease-related deaths [1]. Obesity is characterised by excessive fat accumulation and persistent inflammatory response that may interfere with an optimal state of health. In an obesity state, adipose tissue (AT) inflammation is associated with elevated levels of adipokines. Th1 and Th17 cytokines are also implicated in the development of type 2 diabetics (T2D) [2–4]. These include tumour necrosis factor α (TNF–α) and interleukin (IL)-6, which contribute to insulin resistance, a major feature of T2D, and subsequent vascular dysfunction [5, 6]. Adipose tissue (AT) inflammation is orchestrated by innate lymphoid cells (ILCs) which also regulate various AT metabolic pathways [7]. However, their role in obesity and T2D is yet to be elucidated. ILCs are classified into three distinctive subgroups (ILC1/2/ and 3) based on the transcriptional factors and cytokines they produce. Contradictory findings on the role of ILCs in adipose tissue exist, with the various ILCs subtypes shown to promote inflammation and insulin resistance [8, 9] and, in contrast, improve insulin sensitivity and glucose metabolism [10].

Chronic hyperglycaemia as a consequence of insulin resistance and persistent secretion of inflammatory cytokines from visceral AT is associated with an increased cardiovascular risk in patients with T2D [11]. Pro-inflammatory cytokines which include IL-1/6 and TNF-α together with adhesion molecules exacerbate the inflammatory state and increase the risk of cardiovascular disease (CVD) in T2D [11]. To our knowledge, there are no available systematic reviews that have provided a synthesis of the role of ILCs in T2D and associated CVD risk stratification. Therefore, this systematic review will provide a comprehensive synthesis of available studies reporting on the role of ILCs and their associated analogues such as T helper cells in a T2D state. This study may also provide evidence-based insight into the bidirectional relationship of ILC activation and CVD risk in adults living with T2D.

### Objectives

The objectives of the study are as follows:

1. To determine the activation levels of T helper cells in patients with T2D compared to healthy controls.
2. To determine the levels of ILCs in adipose tissue and peripheral blood of patients with T2D compared to healthy controls.
3. To evaluate the association between ILCs and traditional cardiovascular risk factors in patients with T2D.

## Methods

This systemic review protocol has been prepared in accordance with Preferred Reporting Items for Systematic Review and Meta-Analysis Protocols 2015 guidelines [12].

### Systematic review registration

The protocol has been registered with PROSPERO (CRD42018106159).

### Eligibility criteria

Studies will be included based on the fulfilment of the following criteria.

#### Study design

This systematic review will include randomised clinical trials, observational studies, and case-control studies, with a clearly defined control population.

##### Participants

The systematic review will mainly include studies reporting on adult obese T2D patients (over 18 years older).

##### Intervention

The systematic review will include studies that report the use of oral glucose-lowering drugs.

##### Comparators

T2D diabetic patients on treatment are compared to healthy controls.

##### Outcomes

The study outcomes will include the following:

1. Primary outcomes
  - ILC and CD4 T cell activation (reported as mean expression programmed cell death protein 1, CTLA4, major histocompatibility complex class-II (MHC-II), CD44 expression)
  - CVD (reported as OR)
2. Surrogate outcomes
  - The ratio of ILC subtypes (natural killer cells) and CD4 T cell cytokine levels (reported as mean TNF-α, interferon gamma (INF-γ), IL-5, IL-6, and IL-13 levels)
  - Cardiovascular risk (cholesterol, low-density lipoproteins, triglycerides)

#### Search strategy

A search strategy will be developed using Medical Subject Heading (MeSH) with keywords that include type 2 diabetes mellitus, innate lymphoid cells, natural killer cells, and T helper cells. The search strategy will be used to search the available electronic databases such as MEDLINE, Embase, Cochrane Collaboration, and ClinicalTrials.gov. The search strategy will be developed in consultation with a subject librarian. The systematic review will include studies published between the year 2000 and April 2019. The search will start from 2000 because ILCs were first discovered around that time period [7, 13]; however, a grouping of ILCs came in 2009 [14, 15]. The search will be restricted to available full-text without any language restriction. We will use the Mendeley referencing manager (V1.19.10) to remove duplicates. The bibliography of included studies will be screened for additional studies.

#### Selection process

Two independent reviewers (VM and BBN) will conduct the selection procedure. Each reviewer will screen the titles, abstracts, and full texts in contrast to the inclusion criteria. The reviewers will also exclude studies without available full texts and those reporting duplicate data from the same study cohort. The level of inter-rater agreement will be assessed using Cohen’s kappa inter-rater reliability.

## Data management

### Data items

The reviewer (VM and BBN) will develop a data extraction form which will include the following data items: first author’s name, year of publication, country, study design, sample size, age, gender, treatment used, duration of treatment, and experimental techniques used. Two reviewers (VM and TMN) will independently carry out the data extraction and also check for correctness of all extracted data items. The other author (BBN) will be consulted for arbitration in case of any disagreements.

### Data simplification

Studies that report on various treatment interventions will be combined into a single group. In addition, studies reporting similar effect measures for ILC activation as well as T cell activation will be grouped.

### Risk of bias in individual studies

To assess the potential risk of bias, the Downs and Black checklist will be used [16]. Two reviewers (VM and BBN) will make independent judgments based on the four domains of the tool: reporting bias (10 items), external validity (3 items), internal validity (6 items), and selection bias (7 items). The scores will be rated as excellent (25–26), good (20–24), moderate (14–19), poor (11–13), and very poor (< 10). In case of disagreements, PVD will be consulted to arbitrate.

### Data synthesis

If an efficient number of included studies are homogeneous in terms of study design and participant characteristics, we will use Review Manager V.5 to conduct a meta-analysis using a random effects model. Cochran’s *Q* and the *I*^2^ statistic will be used to assess statistical heterogeneity between the included studies [17]. An *I*^2^ value of > 25 will be considered substantial heterogeneity [18]. To explore the sources of heterogeneity within the included studies, a subgroup analysis and meta-regression comparing the study estimates from different study-level characteristics will include the age, the gender of the study population, the study design and quality, the type of intervention, and the reported effect measure of ILC and T helper activation.

### Sensitivity analysis

We will assess the potential sources of heterogeneity in studies with an *I*^2^ > 50 by performing a sensitivity analysis and by omitting studies that are deemed as of high risk based on the risk of bias.

### Strength of evidence

Two reviewers (VM and BBN) will assess the strength of evidence on the included studies using the Grading of Recommendations Assessment, Development and Evaluation approach (GRADE) [19]. The quality of evidence will be assessed across the domains of risk of bias, consistency, directness, precision, and publication bias. The evidence of each outcome will be rated as either high, moderate, low, or very low. The GADEpro tool will be used to create a summary of findings (SoF) table.

## Discussion

This systematic review and meta-analysis will provide a comprehensive synthesis of studies reporting on the activation levels and function of ILCs and T helper cells in T2D. This is part of a big study assessing the functional role of T cell activation and other inflammatory markers in metabolic diseases, especially those involving T2D and associated CVD complications [20, 21]. This review will also provide insights into the effects of anti-hyperglycaemic drugs on ILCs and T helper cells in T2D. This will therefore allow the identification of evidence gaps and also provide an evidence-based knowledge of associations between immune activation and T2DM.

## Abbreviations

ILCs: Innate lymphoid cells
MeSH: Medical Subject Heading
NK cells: Natural killer cells
T2D: Type 2 diabetes mellitus
TNFα: Tumour necrosis factor alpha
